# Supplementary material for: A review of raptor carcass persistence trials and the practical implications for fatality estimation at wind farms
Source: PeerJ. 2022 Nov 15;10:e14163. doi: 10.7717/peerj.14163 (PMC9673768; doi:10.7717/peerj.14163)
Supplement: Supplemental Information 8 [file peerj-10-14163-s008.docx]

## Supplementary file S8: Mock GenEst dataset for fatality estimation

GenEst requires five files for fatality estimation: carcass persistence, which forms the basis for this paper, searcher efficiency, density-weighted proportion, search schedule and carcass observations (for discussion of each of these parameters, see Simonis et al., 2018).

We generated a plausible mock dataset based on a 12-turbine wind farm with data relevant to estimating raptor fatalities.

For searcher efficiency, we used the same values as in the example GenEst ‘Wind---Cleared plots, bats + birds’ datasets for large carcasses (Simonis et al., 2018), where p=0.661 (0.601-0.716) and k=0.669 (0.559-0.763).

The search schedule was all 12 turbines being searched on a single day, starting on the first day of the year (i.e. January 01). Two separate files were created with a 14-day (i.e. January 1, January 15, January 29, etc.) or 28-day (i.e. January 1, January 29, February 26, etc.) search frequency.

The density-weighted proportion for all turbines was set at 0.55, the same value as in the example GenEst ‘Wind---Cleared plots, bats + birds’ datasets for large carcasses (Simonis et al., 2018).

For carcass observations, we determined that one fatality would occur on the first day of each month (i.e. January 1, February 1, etc., with 12 carcasses for the year), which would be found on the next survey. Two files were created where carcass observations aligned with the relevant search dates for the 14-day or 28-day search interval.
